# Research on the function of *CsMYB36* based on an effective hair root transformation system

**DOI:** 10.1080/15592324.2024.2345983

**Published:** 2024-04-30

**Authors:** Xi Shen, Ting Yang, Yalin Du, Ning Hao, Jiajian Cao, Tao Wu, Chunhua Wang

**Affiliations:** a College of Horticulture, Hunan Agricultural University, Changsha, China; b Key Laboratory for Evaluation and Utilization of Gene Resources of Horticultural Crops (Vegetables, Tea, etc.), Ministry of Agriculture and Rural Affairs of China, Changsha, China

**Keywords:** Cucumber, *Agrobacterium rhizogenes*, hair roots system, *CsMYB36*, Casparian strip

## Abstract

The hairy root induction system was used to efficiently investigate gene expression and function in plant root. Cucumber is a significant vegetable crop worldwide, with shallow roots, few lateral roots, and weak root systems, resulting in low nutrient absorption and utilization efficiency. Identifying essential genes related to root development and nutrient absorption is an effective way to improve the growth and development of cucumbers. However, genetic mechanisms underlying cucumber root development have not been explored. Here, we report a novel, rapid, effective hairy root transformation system. Compared to the in vitro cotyledon transformation method, this method shortened the time needed to obtain transgenic roots by 13 days. Furthermore, we combined this root transformation method with CRISPR/Cas9 technology and validated our system by exploring the expression and function of *CsMYB36*, a pivotal gene associated with root development and nutrient uptake. The hairy root transformation system established in this study provides a powerful method for rapidly identifying essential genes related to root development in cucumber and other horticultural crop species. This advancement holds promise for expediting research on root biology and molecular breeding strategies, contributing to the broader understanding and improvements crop growth and development.

## Introduction

Cucumber is an economically important vegetable crop. Significant cucumber research milestones, such as completing cucumber genome sequencing, were achieved as early as 2010.^1^ In addition, comprehensive investigations, including root-specific transcriptome and proteome analyze, expressed sequence tag (EST) profiling, large-scale cDNA sequencing initiatives, and genome-wide association studies (GWASs), have been carried out in cucumber.^2^ Substantial breakthroughs have primarily concentrated on cucumber plant architecture, fruit shape, variation, and sex differentiation.^3,4^ In contrast, genes essential for root development and nutrient absorption are rare. Although a stable genetic transformation system mediated by *Agrobacterium tumefaciens* (*A. tumefaciens*) for cucumbers has been established, challenges remain in the direct and stable transformation of unknown genes. These challenges include long transformation periods, high costs, and uncertainties. Compared to *A. tumefaciens*-mediated transformation systems, *Agrobacterium rhizogenes*-mediated hairy root transformation exhibits increased speed and enhanced transformation frequency.

Hairy root transformation systems have been established in cucumber to study salt stress and root hair development.^5,6^ However, the transformation frequency of the *in vitro* cotyledon transformation protocols require 34–37 days.^6^ Although the *in vivo* puncture hypocotyl transformation method is simple and rapid for obtaining the transgenic hair roots, it does not enable subculturing preservation and propagation of transgenic root materials. This impedes the high-throughput characterization of gene function, underscoring the need for a rapid, straightforward, and exceptionally efficient technique.

*MYB36* belongs to the R2R3 subfamily of the MYB family.^7,8^ *AtMYB36* specifically expressed in the endodermis of roots,^9,10^ the protein directly regulates the expression of *CASP1*, *PER64*, and *ESB1*, which are the essential genes involved in the formation of the Casparian strip.^11^ In rice, knockout of all three *OsMYB36s* led to the complete absence of the Casparian strip in the endodermal cell layer.^12^

In this study, we describe a rapid, simple and highly efficient *A. rhizogenes*-mediated hairy root transformation system that enables the rapid characterization of gene function and gene editing in cucumber. The entire workflow of this system takes only 22 days. This system was also utilized for the expression of a reporter gene β-Glucuronidase (GUS) and for the validation of CRISPR/Cas construct activity. Targeted mutations, including those in the targeted gene *CsMYB36*, were detected in primary and propagated transgenic hairy roots. This simple and efficient method for cucumber hairy root transformation can also facilitate the study of root biology in plant species other than cucumber.

## Materials and methods

### Plant material

**Figure 1.**
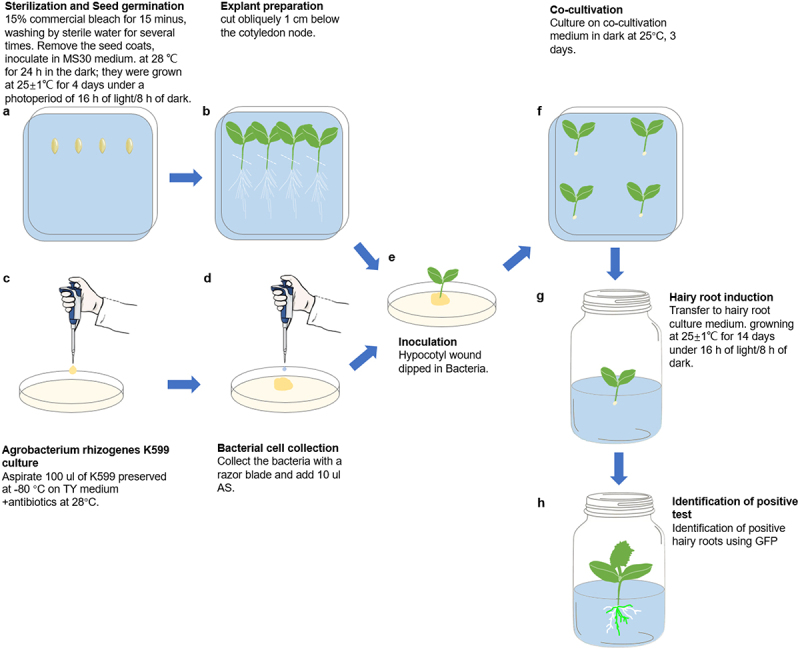
Schematic representation of *agrobacterium rhizogene*s mediated *in vivo* hypocotyl transformation. (A) Sow the sterilized seeds in the MS30 medium. (B) Explant preparation. The white dashed line represents the position of the cut. (C) Culture *agrobacterium rhizogene*s strain K599. (D) Collect bacteria and add AS. (E) Dip bacteria in the wound area. (F) The infected explants on co-cultivation medium. (G) Hairy root induction. (H) hairy root formation at 14 days after co-cultivation, *n* ≥ 30.

Seeds of inbred CU2 cucumber (*Cucumis sativus* L.) (Provided by Hunan Academy of Agricultural Sciences) were water-bathed at 55°C for 30 min after removing seed coats, surface sterilized in 75% alcohol for 30 s and then in 7.5% sodium hypochlorite for 15 min, then washed 4–5 times in sterile water^5^ (Extended Data Figure S1A). Unwounded seeds were placed on MS30 medium (Table 1) for 1 day in the dark (Figure 1(a) and Extended Data Figure S1B), and then grown at 25 ± 1°C and 16 h light/8 h dark for 4 days to obtain the true leaf unfolding explants (Figure 1(b) and Extended Data Figure S1C). *in vitro* cotyledon transformation method, only need 2 days in the dark.^5^

**Table 1.** The list of media used in cucumber hairy root transformation.

Medium	Composition or references
MS30	MS,^13^ 30 g/L sucrose and 6 g/L Phytogel; pH 5.8
Co-cultivation	MS30, 100/200/300/400 μM AS, 3 g/L Phytogel; pH 5.8
Hairy root culture	MS30, 300 mg/L cefotaxime, 3 g/L Phytogel; pH 5.8

### Vector construction for hairy root assay

To make β-Glucuronidase (GUS) reporter lines, the promote of *CsMYB36* was amplified by PCR using CU2 cucumber genomic DNA as a template; PCR amplification was performed using a high-fidelity DNA polymerase kit (Novozymes, following the manufacturer’s instructions). The forward and reverse primers contained *Sal*I and *Xba*I restriction intronic sites, respectively (restriction sites underlined in the primer sequences). The PCR fragments were homologously recombined into the restriction endonuclease *Sal*I and *Xba*II (NEB, MA, USA) sites of the pCAMBIA1305 vector, where the *CsMYB36* promoter was placed to drive the expression of the GUS gene. CaMV35S promoter turn on green fluorescent protein (GFP) expression (p35S:GFP), with the backbone derived from pCAMBIA1305. To induce targeted mutations in the *CsMYB36* gene, we used CRISPR/Cas9 system.^14^ The guide RNA (gRNA) was generated using the CRISPR RGEN Tools (http://www.rgenome.net/) and was designed based on exon sequences, with two sites chosen as targets. A 100-fold dilution of pCBC-DT1T2 was used as a template for tetra-primer PCR amplification, which was performed using the Novozymes High Fidelity DNA Polymerase Kit (Vazyme, Nanjing, China), following the manufacturer’s instructions. After purification, the two fragments were assembled into the destination vector pKSE401 using the *Bsa*I (NEB, MA, USA) enzyme site.^14^ The final constructed vector was confirmed by sequencing, named CR-*CsMYB36*. p*CsMYB36: GUS* and CR-*CsMYB36* binary vectors were mobilized into *Agrobacterium rhizogenes* strain K599 and used for cucumber hairy root transformation. The primers used for vectors construction are shown in Supplemental Table S1.

### Agrobacterium rhizogenes mediated in vivo hypocotyl transformation

The transgenic strain utilized was *Agrobacterium rhizogenes* strain K599 (NCPPB2659; WEIDI, Shanghai, China), which carries the pRi2659 Ri plasmid. K599 with the desired binary vector was spread onto the surface of TY (tryptone-yeast extract) solid plate (supplemented with same antibiotics) and incubated at 28°C overnight (Figure 1(b)). Collect bacteria and 10 µl of 0,100,200,300,400 µM Acetosyringone (AS) was added at concentrations and mixed well (Figure 1(c), Extended Data Figure S2A). Cucumber seedlings with unfolded cotyledons (approximately 5 days), the hypocotyls were cut 1 cm below the cotyledon nodes and dipped into the bacterial mixture and placed in co-cultivation medium for 0,3,5,7 days (Figure 1(d–f), Extended Data Figure S2B–D). Finally, they were inserted into the Hairy root culture medium for about 14 days (Figure 1(g,h), Extended Data Figure S3A, B). The *in vitro* cotyledon transformation method according to Nguyen.^5^ 30 seedlings were infested each time and repeated three times. The detail protocol for the *in vivo* hypocotyl transformation is shown in Extended Data 1.

### Fluorescent observation

The GFP fluorescence of cucumber plants was detected using the LeicaM205 FCA (Leica, Wetzlar, Germany) and VILBER NEWTON7.0 Bio plus (Vilber, Paris, France) with excitation at 488 nm, emission at 507 nm.

### Sequence analysis

To Analysis the evolution of MYB36, according to the evolutionary history of the plants, plant species with different evolution stages were chosen, including the dicotyledon plants: *Arabidopsis thaliana* (L.) (AT), *Oryza sativa* L. (Os) and *Cucumis sativus* L. (Cs). The completed amino acid sequences of all plant species were downloaded from Phytozome v12.11.^15^ Homologous genes were used ATMYB36 blast in amino acid sequences of all plant species based on an expected value (E-value) cutoff of 1 × 10^−5^ in the completed amino acid sequences of all plant species. We implemented Blast searches of the complete protein sequences of all species, extracted protein sequences using TBtools program and performed multiple sequence alignment by MEGA11 (https://www.megasoftware.net/).^16^ The approximately maximum likelihood phylogenetic trees were constructed by Phylogeny e program of MEGA11 using Neighbor Joining (NJ) model and Shimodaira Hasegawa test. Structural domain prediction with NCBI CDD (https://www.ncbi.nlm.nih.gov/Structure/bwrpsb/bwrpsb.cgi) followed by visualization with TBtools. See Supplemental Table S2 for the alignments of MYB36 homologs in different species.

### Identification and characterization of induced mutant hairy roots

Genomic DNA was extracted from hairy roots using the Fast Pure Plant DNA Isolation Mini Kit (Vazyme, Nanjing, China). Hairy roots with induced mutations in *CsMYB36* genes were detected by sequencing (Tsingke Biotech Co, Beijing, China). Briefly, the spanning target regions on the *CsMYB36* genes were amplified using flanking specific primers (Supplementary Table S1). The PCR amplifications were conducted using 2 × Rapid Taq Master Mix (Vazyme, Nanjing, China), and then sequencing of PCR products.

### Staining, sectioning and microscopy

CR-*CsMYB36* gene editing hairy roots were obtain based on our established method (Extended Data 1). For lignin staining, Calcofluor White (Sigma-Aldrich) and Basic Fuchsin (Sigma-Aldrich) staining was performed according to the protocol from the Geldner Lab (see http://wp.unil.ch/geldnerlab/resources-and-protocols/protocols/).^17^ Briefly, the gene editing hairy roots were fixed 3–4 cm from the root tip with 4% paraformaldehyde, stained for overnight with 0.2% Basic Fuchsin (Sigma, MO, USA) (dissolved in ClearSee), washed with ClearSee solution for at least 1 h or overnight, and stained for 30 min with the 0.1% Calcofluor White (Sigma, MO, USA) (in ClearSee). After that, roots were embedded in 5% (w/v) agar and 100 μm sections were obtained on a microtome (Leica VT1000S, Germany). The sections were mounted in 50% ClearSee solution and imaged on a confocal microscope with 580-nm excitation and 600- to 615-nm detection for Basic Fuchsin, 405-nm excitation and 415- to 440-nm emission for Calcofluor White with Zeiss LSM710 (Zeiss, Oberkochen, Germany). GUS staining was performed according to Jefferson.^18^ The transgenic roots were fixed in 90% acetone for 20 min followed by GUS wash solution containing 100 mM Na_2_HPO_4_/NaH_2_PO_4_ (pH7.0), 1 mM K_3_Fe(CN)_6_, 10 mM Na_2_EDTA, and 0.1% (V/V) Triton-100. The roots were incubated in staining solution (GUS wash solution and 2 mM X-Gluc) at 37◦C in dark for 3 h. Samples are mounted in HCG (8 chloral hydrate: 3 glycerol: 1 ddH2O) for analysis under microscope (Mshot, Ming-mei Technology Co., Ltd., Guangzhou, China). Root samples at were sectioned (100 μm) with Leica VT1000 S (Leica, Wetzlar, Germany).

### RT-qPCR (Real-time quantitative reverse transcription PCR) gene expression analysis

Total RNA was extracted from the samples using the Fast Pure Universal Plant Total RNA Isolation Kit kit (Vazyme, Nanjing, China) and reverse-transcribed using HiScript® II 1st Strand cDNA Synthesis Kit (Vazyme, Nanjing, China) according to the manufacturer’s instructions.The expression of *CsMYB36* was determined using ChamQ Universal SYBR qPCR Master Mix (Vazyme, Nanjing, China) in a Roche Light Cycler 96 Real-Time PCR instrument (Roche, Basel, Switzerland). *CsActin* was used as an internal control. Three biological replicates were analyzed per sample. The primers used for RT-qPCR are shown in supplemental Table S1.

## Result

### An effective hairy root transformation system for cucumber

**Figure 2.**
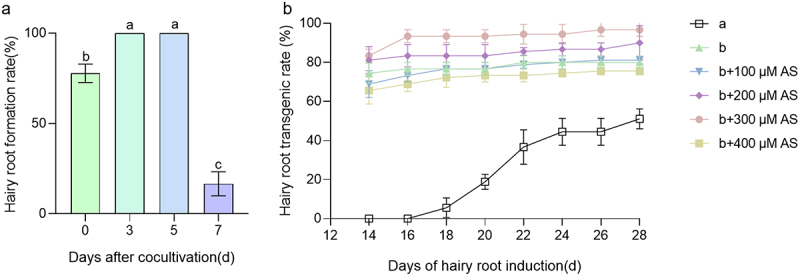
Hairy root formation rate and hair root transgenic rate. (A) Five days old seedlings CU2 were used. Letter a, b and c represented by different letters are very significantly different at the *p* ≤ 0.01 value given. Values are mean ± SD for three independent replicates (*n* ≥ 30). (B) Different treatments hairy root formation at 14 days after co-cultivation. The “a” represents *in vitro* cotyledon transformation method. The “b” represents *in vivo* hypocotyl transformation method. Data are presented as mean ± SD of three replicates, *n* ≥ 30.

The aim of this study was to establish a rapid, simple and highly efficient *A. rhizogenes*-mediated hairy root transformation system in cucumber. Constitutively expressed green fluorescent protein (GFP) was used to screen for positive transgenic hairy roots. The transformation was performed as shown in Extended Data 1. Briefly, the hypocotyls of 5-day old CU2 seedlings were excised under aseptic conditions and only 1 cm of hypocotyl was left. The wounded hypocotyl was inoculated with agrobacteria (K599 strain) (Figure 1(a–h), Extended Data Figures S1-S3). Subsequently, the explants were inoculated into the hairy root culture medium after cocultivation for 0, 3, 5, or 7 days. After a 14-day incubation in hairy root culture, the hairy root formation rates in explants subjected to 3 and 5 days of cocultivation reached 100%, while the hairy root induction rate was 83.34 ± 6.67% after 0 days of co-cultivation. Notably, the percentage of explants sharply decreased after 7 days of cocultivation, with only 6.67% ± 3.34% resulting in browning at the infiltration point of the incision (Figure 2(a)). Thus, 3 days of co-cultivation was sufficient to induce successful hairy root production from the explants, while Nguyen’s method took 5 days.^5^ Moreover, the experimental cycle was shortened by approximately 13 days compared to Nguyen’s method.^5^ This optimized method for cucumber hairy root transformation, with its rapid induction and high efficiency, holds promise for advancing studies in plant genetics and biotechnology.

Acetosyringone (AS) has been shown to permeate *Agrobacterium rhizogene* hairy root cells, activating a sensing mechanism and increasing the affinity between *Agrobacterium rhizogene* hairy roots and plant cells, thereby enhancing transformation efficiency.^19^ To evaluate the effect of AS on the transformation efficiency of cucumber hairy roots, AS was added to the infected plants at final concentrations of 0, 100, 200, 300, and 400 µM. After 3 days of culture, the infected plants were transferred to Hairy root culture medium for an additional 14 days, after which their transformation efficiencies were assessed. Simultaneously, we employed the *in vitro* cotyledon transformation method for comparison. After 14 days of induction culture, the *in vitro* cotyledon transformation method resulted in minimal positive roots. In contrast, the transformation efficiency of the isolated hypocotyl transformation method peaked at 83.33 ± 3.33% with the addition of 300 µM AS. After 28 days of induction culture, the transformation efficiency of the isolated cotyledon transformation method for positive hairy roots was 51.11 ± 5.09%. Remarkably, the *in vivo* hypocotyl transformation method achieved the highest transformation efficiency for positive hairy roots, reaching 96 ± 3.33% with 300 µM AS supplementation, demonstrating a remarkable conversion rate of 96%. Thus, the *in vivo* hypocotyl transformation method features a straightforward procedure, a short cycle, and notably high transformation efficiency. These findings not only improve the understanding of the role of AS in enhancing transformation processes but also highlight the practical advantages of the *in vivo* hypocotyl transformation method for achieving efficient and rapid cucumber hairy root transformation, with potential applications in plant biotechnology (Figure 2(b)).

### Utilizing optimized root transformation methods to analyze the expression of CsMYB36 in roots

**Figure 3.**
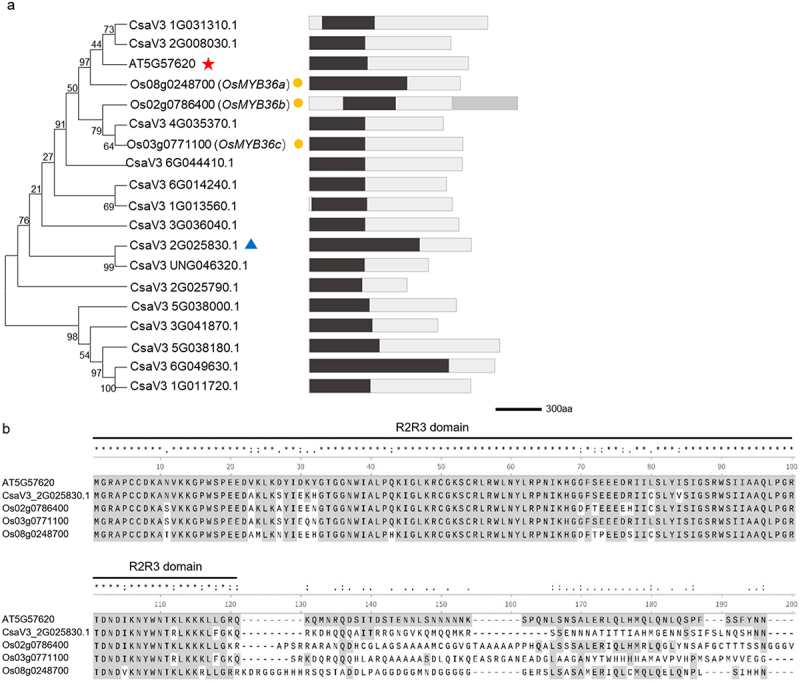
Sequence analysis of MYB36. (A) Phylogenetic tree and structural domain visualization. The red star highlights ATMYB36. The blue triangle highlights CsMYB36. The yellow dots highlight OsMYB36s. The scale bar is the number of amino acid substitutions per site. Black blocks indicate PLN030391 superfamily, and gray block indicate CAP_PR-1. Scale bars, 300 bp. (B) Alignment of amino acid sequences of MYB36 across different plant species. The conserved R2R3-type MYB domain is indicated by the black line.

**Figure 4.**
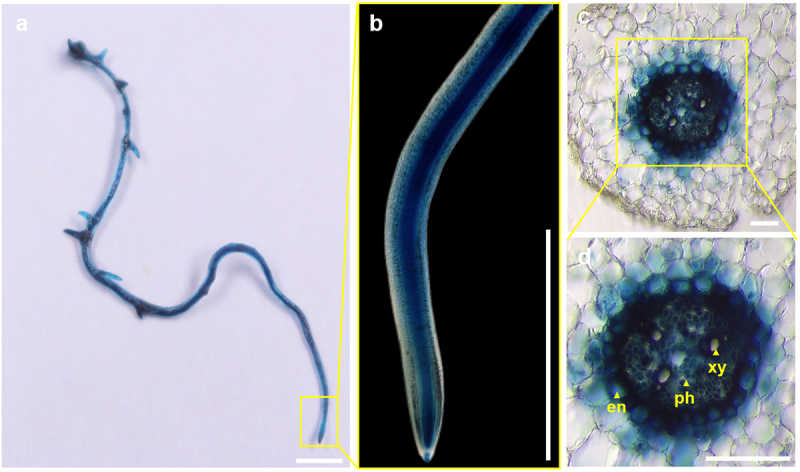
The expression pattern of *CsMYB36* in the cucumber roots. (A) Histochemical localization of GUS activity in cucumber roots transformed with p*CsMYB36: GUS*. Scale bars, 1 cm. (B) Represent the magnified image of the yellow boxed area in (a). Scale bars, 0.5 cm. (C) the cross-sectional of GUS activity in cucumber roots transformed with p*CsMYB36: GUS*. Yellow arrows indicate the cross-sectional structure of cucumber roots. Scale bars, 50 µm. (D) Represent the magnified image of the yellow boxed area in (c). en, endodermis; xy, xylem; ph, phloem. Scale bars, 50 µm. *n* ≥ 5.

Previous studies have shown that MYB36 plays a crucial role in regulating the formation of Casparian strips in Arabidopsis and rice (*Oryza sativa* L.).^11,12^ These results showed that the function of MYB36 in controlling the formation of Casparian strips in plants was likely conserved. Thus, we further explored the function of MYB36 in cucumber roots. By BLAST analysis of the amino acid sequences of *ATMYB36* ^11^ and *OsMYB36a/b/c* ,^12^ we identified 15 genes within the cucumber genome database. Structural domain predictions for these 15 genes revealed a remarkably conserved PLN030391 superfamily domain in the N-terminal region (Figure 3(a)).^20^ The MYB-R2R3 domain, which is integral to this superfamily, resembles ATMYB36 but exhibits substantial divergence in its respective C-terminal region^21^ (Figure 3(b)). Notably, among these genes, *CsaV3_2G025830.1* exhibited significant similarity to the *ATMYB36* protein. In our previous study, we found that *CsaV3_2G025830.1* regulated yellow-green peel color in cucumber and was subsequently named *CsMYB36* .^22^ To investigate the expression pattern of *CsMYB36* in roots, we generated a transgenic strain in which GUS (β-glucuronidase) was initiated by the *CsMYB36* promoter. We used the optimized transformation method to obtain the transgenic hair roots of p*CsMYB36:GUS*. Unlike *AtMYB36* in Arabidopsis, *CsMYB36* exhibited high expression in the whole roots, including the meristematic, elongation, and mature zones in cucumber (Figure 4(a,b)). Furthermore, *CsMYB36* was strongly expressed in endodermal cells and vascular tissues, but weakly expressed in cortical cells adjacent to the endodermal layer in the mature zone (Figure 4(c,d)).

### Utilizing optimized root system and CRISPR/Cas9 system to decipher a function of CsMYB36

**Figure 5.**
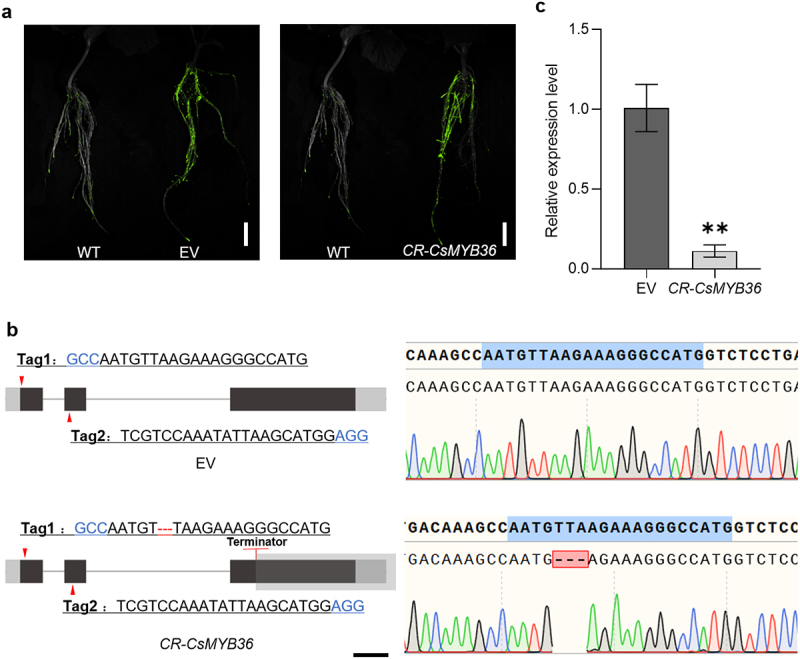
Identification and characterization CR-*CsMYB36* mutant hairy roots. (A) GFP report gene in positive hair root identification. Scale bars, 2 cm. (B) Maps of EV (empty vector) and CR-*CsMYB36*. The red arrows indicate primer binding sites for genotyping. Black blocks indicate coding sequence (CDS), and gray block indicate untranslated region (UTR). Scale bars, 200 bp. (C) Expression of *CsMYB36* in EV and CR-*CsMYB36*. Statistical significance was evaluated by student’ s t test; **p <. 01. n ≥ 5.

**Figure 6.**
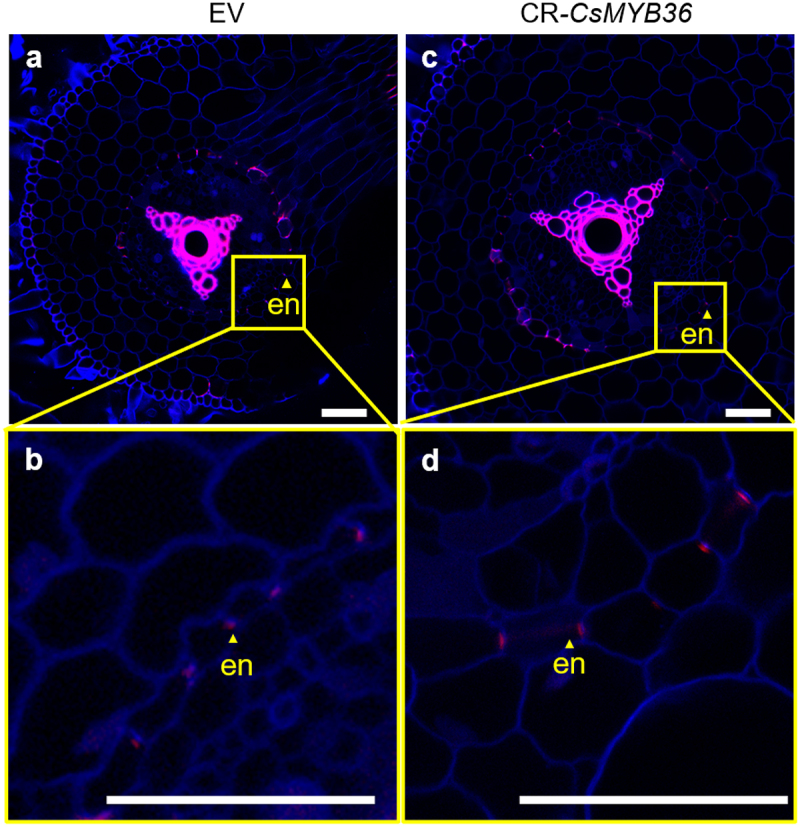
Observations on EV and CR-*CsMYB36* hair root lignin staining. (A–D) roots with 3–4 cm from the apex, EV (A, B) and CR-CsMYB36 (C, D) hair root lignin staining. Magenta and blue show signal of lignin and cellulose, respectively. Scale bars, 100 µm. (B) and (D) represent the magnified image of the yellow boxed area in (A) and (C), magenta light dots show signal of lignin deposited in the endodermis. Scale bars, 100 µm. en, endodermis. *n* ≥ 5.

To investigate the potential regulatory role of CsMYB36 in Casparian strip formation within cucumber roots, we employed an optimized hairy root transformation system combined with CRISPR/Cas9 technology to induce targeted mutations in CsMYB36. We identified many edited lines including one with three base deletions, which led to a frameshift mutation (Figure 5). We conducted sectioning and lignin staining of the propagated hairy roots to assess the formation of the Casparian strip. The Casparian strip diffusion barrier in plants is made of a lignin polymer.^23^ If CsMYB36 regulates the formation of Casparian strips in cucumber roots, then knocking out CsMYB36 will result in the absence of lignification in the endodermal layer of the roots. To test this hypothesis, we stained the roots with basic fuchsin which is a fluorescent lignin dye.^17^ Compared with the WT, the knockout of *CsMYB36* did not exert a significant influence lignin deposition in the cucumber roots. These results suggest that CsMYB36 likely does not contribute to the formation of the Casparian strip in the root endodermis (Figure 6).

## Discussion

The cucumber genome contains 23,248 protein-coding genes.^24^ However, only a few of these genes have been functionally validated. The genes whose functions have been elucidated primarily focused involve the above-ground tissues of the cucumber. Such as genes related to fruit shape, plant architecture, and fruit trichome development have been identified.^25–27^ Cucumber roots are a typical shallow root, but little is known about the genes involved in the development. The stable genetic transformation of cucumber mediated by *Agrobacterium tumefaciens* is time-consuming and labor-intensive, making this method unsuitable for high-throughput functional genomics studies. Therefore, the use of *A. rhizogenes* to induce the transformation of hairy roots represents a novel approach for the functional analysis of cucumber root development genes. This approach can quickly and preliminarily explore the expression and function of such genes in roots.

Previous studies have indicated that hairy roots can be induced from two cucumber explant types, such as *in vitro* cotyledon and *in vivo* hypocotyl. Transgenic hairy roots have been used to investigate root hair development, salt tolerance and scion root-stock communication.^5,6^ This approach has been demonstrated to be effective for the analysis of gene function within cucumber hairy roots. However, based on the previously reported protocols, we believe that the methodology for the cucumber hairy root system still has potential for optimization. In this study, we achieved a high hairy root transformation frequency by a novel the transformation procedure. We selected hypocotyl obtained from 5-day-germinated seeds as explants. Wounded hypocotyls inoculated and co-cultivated exhibited the highest percentage of positive transformation 83.33% (Figure 2(a)). The addition of a selective AS to the root production medium increased the transformation frequency to 96% (Figure 2(b)) and the process took only 22 days, which was faster than those of other methods.^5^ Moreover, our improved method allows for the propagation of transgenic hairy roots, providing an endless supply of transgenic material for chromatin immunoprecipitation followed by sequencing (ChIP-Seq) and immunoprecipitation – mass spectrometry (IP-MS) analysis. Thus, this study proposes an intuitive, rapid, efficient, and sustainable method for the transient transformation of transgenic cucumber roots.

The Casparian strip, located within the root endodermis, establishes an apoplastic barrier between the vascular tissues and the surrounding ground tissues, thereby regulating the selective absorption of water and nutrients.^28^ Previous studies have shown that MYB36 regulates the formation of Casparian strip in both *Arabidopsis* and *rice* .^9–12,29^ These results indicated that the function of MYB36 in controlling the formation of Casparian strip in plants is likely conserved. In our study, we found that CsMYB36 was expressed in both the vascular bundles and the endodermis of cucumber roots, differing from the expression pattern of AtMYB36 in *Arabidopsis*. Importantly, unlike Arabidopsis *MYB36*，*CsMYB36* did not affect the regulation of lignin deposition in the root endodermis. One reason for this difference is that sub-functionalization of CsMYB36 homologues may have occurred during evolution. We previously identified 15 MYB36 homologous genes in the cucumber genome.^29^ Another reason is that the transcription factors encoded by CsMYB36 may be redundantly involved in regulating root Casparian strip formation. Further research is required to determine that the contributions of CsMYB36, and other homologous CsMYB36 genes to cucumber root development.

This study successfully established an efficient protocol for the induction and transformation of cucumber hairy roots. The hairy root system has been effectively utilized for assessing the expression profiles of transgenes and the functionalities of CRISPR/Cas9 systems over a period of 22 days. Our method provides a potential tool for transgene expression and genome editing studies in *Cucumis* plants as well as other crops such as flowers and fruit trees.

## Supplementary Material

Extended Data1 .docx

Supplementary_Data_1.docx
