# Supplementary material for: Research on the function of CsMYB36 based on an effective hair root transformation system
Source: Plant Signal Behav. 2024 Apr 30;19(1):2345983. doi: 10.1080/15592324.2024.2345983 (PMC11062371; doi:10.1080/15592324.2024.2345983)
Supplement: Extended Data1 .docx [file KPSB_A_2345983_SM3583.docx]

***Protocol for the in vivo hypocotyl transformation***

**Section  I: *Agrobacterium rhizogenes*transformation**

**Section  II:  Hairy Root Transformation**

**Section  III: Media recipes**

**I: *Agrobacterium rhizogenes* transformation**

Adapted from *Agrobacterium rhizogenes* strain K599^1-3^ Chemically Competent Cell product manual (WEIDI, Shanghai, China).

**Competent cells:** *Agrobacterium rhizogenes* strain K599 competent cells (NCPPB2659; carrying pRi2659 Ri plasmid; WEIDI, Shanghai, China)

**Transformation:**

1. The *Agrobacterium rhizogenes* strain K599 competent cells stored at -80°C was left at room temperature or in the palm of the hand for a few moments to partially melt and was inserted into ice when in a mixed state of ice and water.
2. Add 0.01-1 μg of plasmid DNA to each 100 μl of the competent cells and mix by rapidly and vigorously dialing the bottom of the tube by hand or blowing and mixing with a pipette gun, and then place in ice for 5 minutes, liquid nitrogen for 5 minutes, 37℃ water bath for 5 minutes, and ice bath for 5 minutes in that order.
3. After ice bath, take it out to room temperature, add 700 μl of antibiotic-free TY liquid medium, and incubate for 2 hours at 28°C with shaking.
4. Collect the bacteria by centrifugation at 6000 rpm for one minute, leave about 100 μl of the supernatant to be gently blown and resuspended, and then spread on the TY with agar containing appropriate  antibiotics, and then inverted and placed in an incubator at 28℃ for 2 days.

**II:  Hairy Root Transformation**

Adapted from Nguyen’s^4^ and Fan ’s^5^ methods.

NOTE: For any of these steps, you should be working inside a Laminar Flow Hood to keep a sterile environment.

1. **Seed sterilization and germination (5-7 days)**

Materials: forceps, ddH_2_O, 75% EtOH, 7.5% sodium hypochlorite, 250 ml conical flask, 10 x 10 cm sterile, disposable petri with MS30 medium and growth chamber (25 ± 1℃ 16:8 photoperiod).

Operating procedure: Seeds of a local cucumber (*Cucumis sativus* L.) CU2 (Provided by Hunan Academy of Agricultural Sciences) were water-bathed at 55℃ for 30 min After removing seed coats, surface sterilized in 75% EtOH for 30s and then in 7.5% sodium hypochlorite for 15 min, then washed 4–5 times in sterile water7. Unwounded seeds were placed on MS30 medium for 1 day in the dark, and then the grown at 25 ± 1℃ and 16 h light /8 h dark for 4 days to obtain the true leaf unfolding explants (**Extended Data Figure 1**).


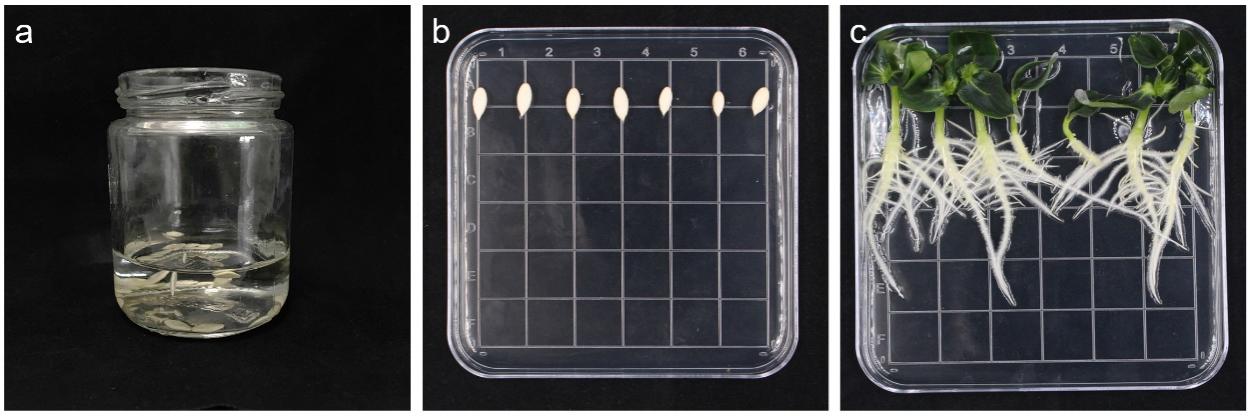


**Extended Data Figure 1. Seed sterilization and germination** (a) Seed soaking and sterilization. (b) Sowing in MS30 plates. (c) The 5-day-old seedlings. n≥30.

1. **Preparation of *Agrobacterium rhizogenes* cells (1 day)**

Materials: 60 x 10 mm sterile, disposable petri TY plates with agar containing appropriate antibiotics and 28°C incubator.

Operating procedure: Take a 100 µl *Agrobacterium rhizogenes* cells (Carrying transformation plasmids) to uniform coating a 90 ×100 mm petri dish with agar containing appropriate antibiotics and culture 24 h in 28°C.

1. **Transformation and Co-cultivation (3 days)**

Materials: surgical knife and blade, forceps, 300 µM AS, 10 x 10 cm sterile, disposable petri with MS30 + Acetosyringone (AS)plates and growth chamber (25 ± 1℃, 16:8 photoperiod)

Operating procedure: Collect bacteria by knife and 10 µl of AS was added at concentrations and mixed well. Cucumber seedlings with unfolded cotyledons (approximately 5 days), the hypocotyls were cut 1 cm below the cotyledon nodes and dipped into the bacterial mixture and placed in 10 x 10 cm sterile, disposable petri with MS30 + AS plates at 25 ± 1℃ and dark for 3 days (**Extended Data** Figure 2).


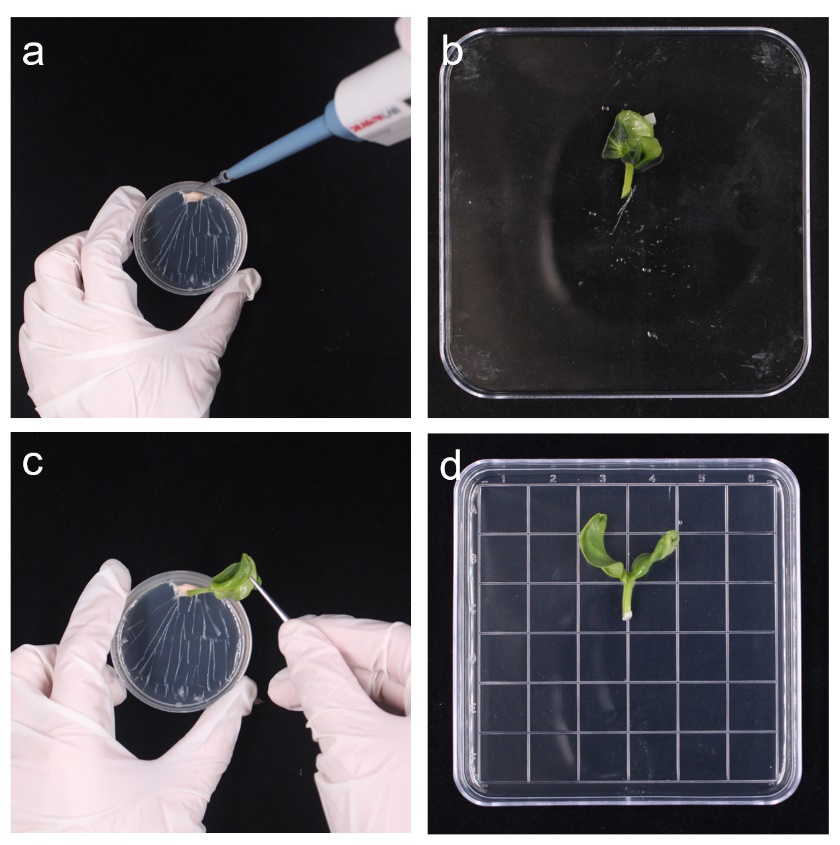


**Extended Data Figure 2. Seed sterilization and germination.** (a) Prepare *Agrobacterium rhizogenes* for infection. (b) Prepare explants for infection. (c) Infect explants. (d) Transfer explants onto co-cultivation plates. n≥30.

1. **Selection and Root Induction (14-21 days)**

Materials: forceps, 82 x 67 mm tissue culture bottle with MS30 + cefotaxime + antibiotic plates and growth chamber (25 ± 1℃ 16:8 photoperiod).

Operating procedure: Insert the wound of the co cultured explant into the 82 x 67 mm tissue culture bottle with MS30 + cefotaxime + antibiotic plates. at 25 ± 1℃ and 16:8 photoperiod for14-21 days (**Extended Data** Figure 3).

.


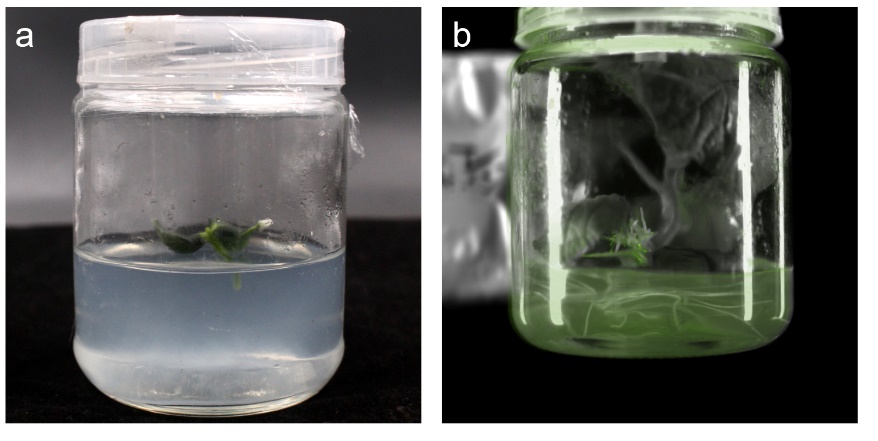


**Extended Data Figure 3. Selection and Root Induction.** (a) After co-cultivation transfer explants onto MS30 + cefotaxime + antibiotic plates. (b) Induce hair roots after 14 days of growth detecting GFP. n≥30.

1. **Subclone the hairy roots (14-21 days)**

Materials: surgical knife and blade, forceps, 10 x 10 cm sterile, disposable petri plates with MS30 + cefotaxime + antibiotic plates and growth chamber (25 ± 1℃ 16:8 photoperiod).

Operating procedure: Once roots are at least 1.0 cm long, roots can be excised from the explant for amplification. Transfer to individual 10 x 10 cm sterile, disposable petri plates with MS30 + cefotaxime + antibiotic plates (**Extended Data** Figure 4.)


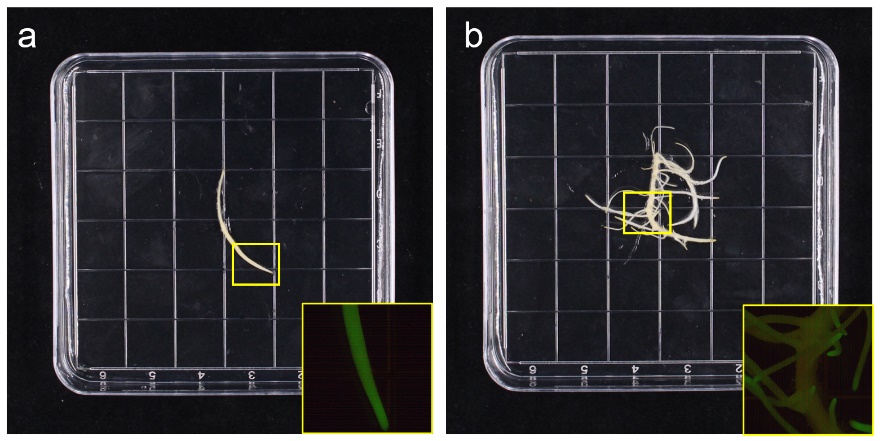


**Extended Data Figure 4. Subclone the hairy roots.** (a) Transfer Hairy roots onto MS30 + cefotaxime + antibiotic plates. (b) Hairy roots after 14 days of growth. Boxes represent GFP detection. n≥30.

**III: Media**

**TY(1 L)**

Materials: 5 g tryptone, 3 g yeast extract, 10 ml 1 M CaCl_2_ and add to 1 L ddH_2_O.

Operating procedure: Place all ingredients (slowly) in 1 L beaker with spin bar. Add ~900 ml ddH_2_O to dissolve ingredients completely. Bring volume up to 1 L. Adjust pH to 7.0 with 1 M NaOH. After complete dissolution, sterilize at 121℃ for 20 min at high temperature. If preparing TY solid culture medium, add 15 g agar.

**MS30 (1 L)**

Materials: 4.43 g MS (Murashige & Skoog), 30 g sucrose, 3 g Phytogel and add to 1 L ddH_2_O.

Operating procedure: Fill a 1L beaker with ~ 900 mLddH_2_0. Add MS and sucrose in a beaker. Adjust pH to 5.8 with 1 M KOH. Add phytogel and Bring volume up to 1 L. After complete dissolution, sterilize at 121℃ for 20 min at high temperature. Allow to cool to 65℃ before adding antibiotics if needed (Plant selection as per your construct, 200 mg/L cefotaxime for killing the *Agrobacterium rhizogenes*).

**Reference**

1 Savka, M. A. Induction of Hairy Roots on Cultivated Soybean Genotypes and Their Use to Propagate the Soybean Cyst Nematode. *Phytopathology*, doi:10.1094/phyto-80-503 (1990).

2 Cho, H.-J., Farrand, S. K., Noel, G. R. & Widholm, J. M. High-efficiency induction of soybean hairy roots and propagation of the soybean cyst nematode. *Planta* **210**, 195-204, doi:10.1007/PL00008126 (2000).

3 Weller, S. A., Stead, D. E. & Young, J. P. W. Acquisition of an Agrobacterium Ri Plasmid and Pathogenicity by Other α-Proteobacteria in Cucumber and Tomato Crops Affected by Root Mat. *Applied and Environmental Microbiology* **70**, 2779-2785, doi:10.1128/AEM.70.5.2779-2785.2004 (2004).

4 Nguyen, D. V. *et al.* An Efficient Hairy Root System for Validation of Plant Transformation Vector and CRISPR/Cas Construct Activities in Cucumber (Cucumis sativus L.). *Front Plant Sci* **12: 770062.**, doi:10.3389/fpls.2021.770062 (2022).

5 Fan, Y. *et al.* A fast, simple, high efficient and one-step generation of composite cucumber plants with transgenic roots by Agrobacterium rhizogenes-mediated transformation. *Plant Cell, Tissue and Organ Culture (PCTOC)* **141**, 207-216, doi:10.1007/s11240-020-01781-x (2020).
