# Supplementary material for: Research on the function of CsMYB36 based on an effective hair root transformation system
Source: Plant Signal Behav. 2024 Apr 30;19(1):2345983. doi: 10.1080/15592324.2024.2345983 (PMC11062371; doi:10.1080/15592324.2024.2345983)
Supplement: Supplementary_Data_1.docx [file KPSB_A_2345983_SM3581.docx]

**Supplementary Table S1.** Primers used in this study

| **Primer’s name** | **Sequence of primers (5’ 🡪 3’)** | **Note** |
| --- | --- | --- |
| p*CsMYB36*-F | TCAAACAGCTTATCGATACCGTCGACCATCAGTGTTTAATAGCACG | Amplifying of the *CsMYB36* gene |
| *pCsMYB36-R* | ACAGGACGTAACATGGATCCTCTAGACCTAATCCAAGAAACTATGAA |  |
| DT1- *CsMYB36-*BsF | ATATATGGTCTCGATTGCATGGCCCTTTCTTAACATGTT | Construction of CRISPR double knockout vector |
| DT2- *CsMYB36-*BsR | ATTATTGGTCTCGAAACCCATGCTTAATATTTGGACGCAA |  |
| DT1- *CsMYB36-*F0 | TGCATGGCCCTTTCTTAACATGTTTTAGAGCTAGAAATAGC |  |
| DT2- *CsMYB36-*R0 | AACCCATGCTTAATATTTGGACGCAATCTCTTAGTCGACTCTAC |  |
| *CsActin*-F | CGCTCTTCTTGCTTTCACCCTT | Primers for RT-qPCR |
| *CsActin*-R | TACCTTGCCTTGGAGTATTTGG |  |
| *CsMYB36*-F | AGCAACTTGACTCCCTTCGG |  |
| *CsMYB36*-R | CTCCACCATTTGGCTGTTGC |  |

The underline sequences are target sequences.

**Supplementary Table S2. The Protein sequence of MYB36s homologs in different species.**

| **Gene ID** | **Protein sequence** |
| --- | --- |
| *CsaV3_1G031310.1* | MVFFLICFQNKTPKREIELFEGKIEEMGRAPCCDKANVKKGPWSPEEDARLKAYIEEHGTGGNWIALPQKIGLKRCGKSCRLRWLNYLRPNIKHGGFSEEEDNIICNLYISIGSRWSVIAAQLPGRTDNDIKNYWNTRLKKKLLGKRKQSNSNNGINSSSSSSNNDDPKELLSNSALERLQLHMQLQGFQNPFSFYTHPQLWPKLHPVQEKIVQTLQSLNNNTQNPNDLQSTTTDTQLLQSHPPKAATDFFIHPQTNTNSISLPCKIPENSDHHQVESSSGHHQVSNFMQVVDGFLEDHNKGEGFMAQSDEQVAEFDWFKEVGGCSKDSLIWWGQNNEHDMRSAASSSNSWDSSSVLQSELMFQDFELGYNL |
| *CsaV3_6G044410.1* | MGRAPCCDKANVKKGPWSPEEDAKLKAYIDQFGTGGNWIALPQKIGLKRCGKSCRLRWLNYLRPNIKHGGFSEEEDNIICSLYISIGSRWSIIAAQLPGRTDNDIKNYWNTRLKKKLLGKQQQAVAARRIPPLKKDDFDKNFNNIIHPSSSLNLNNYEAPYIPNLSTSTQDHQVRDLILKMGGKFYYSDHHHPPSFQFNSPMENQNPLLFQSSNFGELNMEFGASNDGIFHGSVDQNSSGGGEFGDPIMLENFENGLVEDFNNYGMMPSSSTSGESSYSLGEISSLGFNYSDFEASQHQHHHHVIAPAFAHQSTHYSLQ |
| *CsaV3_2G008030.1* | MGRAPCCEKEKVKKGPWSPEEDEKLKSYIHLHGAVSNWIALPHKIGLKRCGKSCRLRWLNYLRPNIKHGGFSEEEDNIICSLFISIGSRWSIIAAQLPGRTDNDIKNYWNTRLKKKLLGTPKQYFSNINKLSSHGNYRDTAQALTNSGIERLQLQMQLHQSTFSFNNASTLWPPMPVGEVKVARTGQLANHNHVDGASCRVGTPMLEGYAMNCRSMKSPTVSSSSTELGRLEGGGGRGGVEFVKGMDGSKESLYWWGYDFDAKSGGATKLWETAASVDVQLEEIFKEFEQFSHSL |
| *CsaV3_2G025830.1*  *(CsMYB36)* | MGRAPCCDKANVKKGPWSPEEDAKLKSYIEKHGTGGNWIALPQKIGLKRCGKSCRLRWLNYLRPNIKHGGFSEEEDRIICSLYVSIGSRWSIIAAQLPGRTDNDIKNYWNTRLKKKLFGKQRKDHQQQAITRRGNGVKQMQQMKRSSENNNATITTIAHMGENNSSIFSLNQSHNNNILPYWPQQQHLPLAPHFNSNLTPFGKPIIENDLSKFGVQFMDQESNLYSSNNNNNGNGNEYGNYLPIMQGQSSSSNSQMVEIENYNNMANYEDPRRVLCGLEFLYGDHHIMSGNNNDLQISSCLPNYEEILLQDLSTTTTTTTQEYGAKFDDLRILDNNAL |
| *CsaV3_4G035370.1* | MGRAPCCDKANVKKGPWSPEEDMKLKSYIEQHGTGGNWIALPQKIGLKRCGKSCRLRWLNYLRPNIKHGDFSEEEDKIICSLYVSIGSRWSIIAAQLPGRTDNDIKNYWNTRLKKKLFGKHYEKQQQQLARRGRKIKSGIGNSMAIVFDNQGIYNNNNNNNQSPFLPELSPALNSPPPPPYASYQPLQFPSNSFSHTAGVIDDLRRTDSVLRFGMEGASSQPVTSYHVGELEKVVYSNTPSFDDGLQFSCDNNGLNLMNDLDWGEMSSLISAPLYPSMII |
| *CsaV3_6G014240.1* | MGRAPCCDKANVKKGPWSPEEDMKLKSYIEQHGTGGNWIALPQKIGLKRCGKSCRLRWLNYLRPNIKHGDFSEEEDKIICSLYVSIGSRWSIIAAQLPGRTDNDIKNYWNTRLKKKLFGKHYEKQQQQLARRGRKIKSGIGNSMAIVFDNQGIYNNNNNNNQSPFLPELSPALNSPPPPPYASYQPLQFPSNSFSHTAGVIDDLRRTDSVLRFGMEGASSQPVTSYHVGELEKVVYSNTPSFDDGLQFSCDNNGLNLMNDLDWGEMSSLISAPLYPSMII |
| *CsaV3_6G014240.1* | MGRAPCCDKANVKKGPWSPEEDSKLKHHIETYGTGGNWISLPQKAGLRRCGKSCRLRWLNYLRPNIKHGEFSAEEDRIICTLFATIGSRWSIIAAQLPGRTDNDIKNYWNTKLKKKLMMGMVSSSSTANTTHCAPQAEVIRKIETLSPKPHQNHSFEYQPFSNVSYEPLLSTSYSIPLFNYCNNPISSCLVTNPFDQNNNFYETLKPNQMGFEFDELEYGGLNSNSFNNGLLFDEKMKMMQQNNNGNEGYNSNYVEGNTMMMKEEEEMMEQKREEEEEEEEQRSTK |
| *CsaV3_3G036040.1* | MGRTPCCDKANVKKGPWSPEEDAKLKDYIQKHGTGGNWISLPQKAGLKRCGKSCRLRWLNYLRPDIKHGDFSEEEDTIICNLYTTIGSRWSVIAAQLPGRTDNDIKNYWNTKLKKKLTSPHGMLPQFIQPSSTNSSSSSSSPSSYSNNGPLISTLLEPISFSSNLLLNPTTTTTNNNATPLFNHQAVSQDHQSFMMSTMVGGENNYHVKLGDQRSLLVFGGDQGSCSSSDAEYGGGIGVEEKRRSLSSSNMSFVEWSRVVNGWNNNEKQLEDQGMWNNNSNMENNYSPFMDYGLEEIKQLISSSNCTTNVLF |
| *CsaV3_UNG046320.1* | MGRAPCCDKASVKRGPWSPQEDATLTAFIHKHGTGGNWIALPHKAGLNRCGKSCRLRWLNYLRPDIKHGGFTQEEDNVICSLYSTIGSRWAVIASQLPGRTDNDVKNHWNTKLKKKFNSSFSKPPNPIVVVTDPPRYSATSTANSLKLHDDHEHNINTNFNMFSSHPPFDQIPMLNNNNNPIIPELSTSSSSISLPIEQNHLEFSGPSSDEAADILQSYFHGVYENNNQYPYEDDVMDGFDVSNYMGL |
| *CsaV3_2G025790.1* | MGRAPCCEKGKVKKGPWSPDEDAILKSYVETHGIAGTWIALPTKAGLKRCGKSCRLRWLNYLRPNIKHGEFTEEEDNVIFNLFNQYGSRWSTIASQLPGRTDNDVKNHWNTKLKKKKKKLFLAAKTQSHLHSQSPLLHTQETDFTEIINHTPISCIAALDNSVDQFQIPTVSEPGAGSDSRDQWSSMEPWGCDFPADMICSMLF |
| *CsaV3_5G038180.1* | MGRSPCCDKVGLKKGPWTPEEDQKLLDYIKEHGHGSWRALPSKAGLQRCGKSCRLRWINYLRPDIKRGKFSLQEEQTIIQLHALLGNRWSAIATHLPKRTDNEIKNYWNTHLKKRLTKMGIDPVTHKPKNDALLTADGHSKSAANLSHIAQWESARLEAEARLVRESRLRTSTTTASTTNPPSPHLLTKPEPPPAIPSRSLDALKPWNHGLGRGITGGDHHLESPTSTLTFSENVRTVMGTGSGGENSIAMIEFVGSSSSCHEWKEEGFGNSSSQLQEDHHQYKEGFENSMSFTSHEMTLSIEATTTTWTSESLRSSTRDHHHHHHHNHNNNNNNDIVEEGFTNLLLNNNVDERNHSDAGGGEESENGSGSDYYEDNKNYWNSILNLVNSSPSDSSIF |
| *CsaV3_3G041870.1* | MGKPPCCDKSNVKRGLWTAEEDAKILAYVSNHGVGNWTLVPKKAGLNRCGKSCRLRWTNYLRPDLRHDSFTPQEEDLIIKLHQAIGSRWSVIAKQLPGRTDNDVKNYWNTKLRKKLLKMGIDPITHKPFSQILFDYGSISSLQTTPKPLMGPFNKTLTPTTTMAKSQQPFSSGTNMGEQFQFQTPKKPYILKEQPTSSSCSSSSINGSETMFHLGSSSGHSNGIEPNLQRCEGSSSDPSFDSFVDALLEQDFEIKGSFPEILDECFDY |
| *CsaV3_6G049630.1* | MGRTPCCEKEGLKKGPWTPEEDQKLIDYIQKHGYGNWRTLPKNAGLQRCGKSCRLRWTNYLRPDIKRGRFSFEEEETIIQLHSILGNKWSAIAARLPGRTDNEIKNYWNTHIRKRLLRMGIDPVTHNPRLDLLDLSSILGSSFYNNNSPNSQMNNFSRLIGIHNSTVNPEVLRFANSFIASNNLSQNPNFLLQNIDQNQEQYSQMIISQLQLQQQQQSHHQIDQSSALPPLHEVSAGCSPSTTTSYGGEPPYYHSSGHQLLFPSSSNFTTDFYSQNCQHPSDKMPSINNNLNGFNYSSLEEFQSYDLYGSHEEREREQEQKQFHEQIMEASPETSTLNSSPTPLNSNSTYFSTANGNGTDQDDRESYCSQIFKFEFSDFLDVNPAFM |
| *CsaV3_1G011720.1* | MGRTPCCDKNGLKKGPWTPEEDQKLIDYIQKNGHGNWRTLPKNAGLQRCGKSCRLRWTNYLRPDIKRGRFSFEEEETIIQLHSVLGNKWSAIATRLPGRTDNEIKNYWNTHIRKRLLRMGIDPVTHSPRLDLLDLSSILRSTLYNSSQMNLSSLLGVQPLVNPELLKLAASLMSSERKNPSFSPQNSSHTTTATIQFSNPQLQMQVPMQEIVQFPSQVVEPNIASELNDDQWGNGQLNNFDLATGSFEYCGLDQQQQAGAIAVDNSYETATFNFHNGNGNGNNFSLGSVLSSPCSSSPTQMNSNSTYFTSPTEDERESYCSQILNFEISDIFDEPFI |
| *CsaV3_5G038000.1* | MGRPPCCDKEGVKKGPWTPEEDIVLVSYIQQHGPGNWRAVPSNTGLLRCSKSCRLRWTNYLRPGIKRGNFTDQEEKMIVHLQALLGNRWAAIASYLPQRTDNDIKNYWNTHLKKKLRKVQVRSNSYGDTSKQGMARGQWEKRLQTDIRMARQALWEALTPQTSTTDFSELLLNPSSLGCFSTKQAQAQAQAQAQAQAQTQAQATAYASSTENIAKLLTGWMKTPAKTEVKTESSGGSGGDGGGDGEFRSILRYECYENGNTCENHEEGMNCEGLPFSMIEKWLLEESGGAQPRDCELNDIGILDENI |
| *CsaV3_1G013560.1* | RQKKMGRSPCCDKSNVKKGPWSPEEDAKLKDFIDKNGTGGNWISLPQKAGLKRCGKSCRLRWLNYLRPNIKHGGFTDEEDRIICTLFSTIGSRWSIIATQLPGRTDNDIKNHWNSKLKRKLIEMEKKAQITTSRTIPSSHNHHHHQIFSSFSSQPLSSLINKHDDYFNDINYSNYNLSSPPTTIKSFESLISSIPSNWSNNNNNISPSPCLVQTQETTNLLSSNSLMYNHHNPLVQMKESCLLGFGSEGSCSSSTSDGRSYTQINTTSGIMGFQNNNNNNNPNYYYYYSDNIMNYINHX |
| *AT5G57620*  *(ATMYB36)* | MGRAPCCDKANVKKGPWSPEEDVKLKDYIDKYGTGGNWIALPQKIGLKRCGKSCRLRWLNYLRPNIKHGGFSEEEDRIILSLYISIGSRWSIIAAQLPGRTDNDIKNYWNTKLKKKLLGRQKQMNRQDSITDSTENNLSNNNNNKSPQNLSNSALERLQLHMQLQNLQSPFSSFYNNPILWPKLHPLLQSTTTNQNPKLASQESFHPLGVNVDHQHNNTKLAQINNGASSLYSENVEQSQNPAHEFQPNFGFSQDLRLDNHNMDFMNRGVSKELFQVGNEFELTNGSSWWSEEVELERKTTSSSSWGSASVLDQTTEGMVMLQDYAQMSYHSV |
| *Os08g0248700 (OsMYB36a)* | MGRAPCCDKATVKKGPWSPEEDAMLKNYIEEHGTGGNWIALPHKIGLKRCGKSCRLRWLNYLRPNIKHGDFTPEEDSIICSLYISIGSRWSIIAAQLPGRTDNDVKNYWNTKLKKRLLGRRKDRGGGHHHRSQSTADDLPAGGDGGMNDGGGGGGERSLSASAMERIQLCMQLQELQNPLSIHHNPLLSHQWPSKATIDDQNHNNVTVAEHGMSSSVSDHHRLDGQQLESGAGAAAMQQASPSSGGENSNVVVAIEAELQELLYAGGGAIVDGGAPPQGDVDWWSYDQGKQSPVTCWDFTPETSSIFQDYATVYDI |
| *Os03t0771100*  *(OsMYB36b)* | MGRAPCCDKASVKKGPWSPEEDAKLKSYIEQNGTGGNWIALPQKIGLKRCGKSCRLRWLNYLRPNIKHGGFSEEEDRIILSLYISIGSRWSIIAAQLPGRTDNDIKNYWNTRLKKKLFGKQSRKDQRQQQHLARQAAAAASDLQIKQEASRGANEADGLAAGANYTWHHHHAMAVPVHPMSAPMVVEGGRVGDDVDESIRKLLFKLGGNPFAASPAPPCIPPPPMYEEAPSFVPPLAHGVPLNEGGMQCSSVLPALELDENFHFNHVKLDGLECLFGMGDHQNMRWNEVSPLVCPNNAVASSSQGMQQYCLVEEPADLGMQ |
| *Os02g0786400 (OsMYB36c)* | MHPLTAAAKHAITIPPPPPPAAAASYTSSPSSGTSDPSVLDLSSAEIDDDGDGDDDRAEQQEIKNSKELVMGRAPCCDKASVKKGPWSPEEDAKLKAYIEENGTGGNWIALPQKIGLKRCGKSCRLRWLNYLRPNIKHGDFTEEEEHIICSLYISIGSRWSIIAAQLPGRTDNDIKNYWNTKLKKKLLGKRAPSRRARANQDHCGLAGSAAAAMCGGVGTAAAAAPPHQALSSSALERIQLHMRLQGLYNSAFGCTTTSSNGGGVGVAPPQWPKLEALLPSRPLPAVQPTDASLASGFLDAHNAARRQVGVPPLRWDERLASYAARYAAARSGAGGGCALVHSHGPYGENLFHGSGVGWAPADVVAAWVSRERALYDAASNSCRGGDAAACGHYTQVVWRRTTAVGCALATCAGGRGTYGVCSYNPPGNYVGVRPY |
